# Activity and movement of free-living box turtles are largely independent of ambient and thermal conditions

**DOI:** 10.1186/s40462-018-0130-8

**Published:** 2018-07-19

**Authors:** Adam F. Parlin, Jessica A. Nardone, John Kelly Dougherty, Mimi Rebein, Kamran Safi, Paul J. Schaeffer

**Affiliations:** 1 Department of Biology, Miami University, Oxford, OH 45056 USA; 2 Max Planck Institute for Ornithology, Vogelwarte Radolfzell, Am Obstberg 1, 78315, Radolfzell, Germany

**Keywords:** Daily activity, Ecophysiology, Overall dynamic body acceleration, Resolution

## Abstract

**Background:**

Ectotherms are assumed to be strongly influenced by the surrounding ambient and environmental conditions for daily activity and movement. As such, ecological and physiological factors contribute to stimuli influencing navigation, extent of movement, and therefore habitat use. Our study focused on the intensity of activity (from acceleration data) and extent of movement (from GPS and thread trailing data) of Eastern box turtles (*Terrapene carolina carolina*) in a fragmented landscape near their northern population limit. First, we quantified the thermal performance curve of box turtles using activity as a measure of performance. Second, we investigated ecological factors that could influence activity and movement and characterized the movement as extensive (exploration) and intensive (foraging).

**Results:**

In contrast to previous lab work investigating effects of temperature on activity, we found no relationship between box turtle activity and temperature in the field. Furthermore, box turtle activity was consistent over a wide range of temperatures. Cluster analysis categorized movement recorded with GPS more as intensive than as extensive, while thread trailing had more movement categorized as extensive than intensive. Box turtle activity was higher during the morning hours and began to decrease as the day progressed. Based on the microclimate conditions tested, we found that box turtle movement was influenced by *precipitation* and *time of day*, and activity was most influenced by *absolute humidity*, *ambient temperature*, *cloud cover*, and *time of day*.

**Conclusions:**

Our model ectotherm in this study, the Eastern box turtle, had activity patterns characteristic of a thermal generalist. Sampling resolution altered the characterization of movement as intensive or extensive movement, possibly altering interpretation. More information on the resolution needed to definitively identify foraging and exploratory behavior in turtles is needed. Activity and movement were nearly independent of environmental conditions, which supports the overall interpretation that turtle performance is that of a broad environmental generalist. Future studies of movement of other turtle and reptile species are needed to determine the generality of these findings.

**Electronic supplementary material:**

The online version of this article (10.1186/s40462-018-0130-8) contains supplementary material, which is available to authorized users.

## Background

Animals move through their environment with a suite of inputs modified by ecological and physiological factors to determine navigation, migration, dispersal, foraging, and exploration. Furthermore, the environment needs to be navigable terrain and free of any major geographic barriers. The habitat occupied by a species, whether continuous or fragmented, is patchy and selection hierarchical [1], and as a result movement patterns depend on integration of numerous ecological, physiological, and behavioral variables [2]. Understanding animal navigation throughout their habitat thus requires high resolution measurements of activity, movement, and multiple aspects of ambient conditions [3], optimally with field-based studies of free-living animals.

Free-living ectotherms are also dependent on microclimate conditions being within tolerable physiological limits [4]. Vegetative structure, topography, and geographic barriers are ecological factors that shape the landscape and influence the microclimate conditions available to ectotherms, thus impacting activity and movement. Additionally, the way in which an individual exploits its environment may be altered by extrinsic or intrinsic factors, potentially leading to alteration of the nature of movement in the available habitat [5]. Within these ecological factors, microclimate conditions including temperature, humidity, and rainfall can alter activity and movement [6, 7]. The relevance of microclimate on physiology depends on how each factor impacts performance. One of the most influential ambient conditions impacting ectotherms is temperature. Ambient temperature and environmental conditions must be within tolerable physiological limits for ectotherms, and laboratory observations strongly support a role for temperature on movement performance in many reptiles [8–10] including box turtles [11]. Prior work on box turtle movement and activity in the lab showed a strong thermal dependence of strides per minute, total time stopped and velocity through test temperatures between 10 and 32 °C [11]. However, laboratory studies may not always reflect patterns and processes observed in nature, and thus may not represent what animals are capable of, or choose to do, under natural conditions. For example, our recent work showed that in the field, box turtle movement was not correlated with body temperature [12].

Further, the physiologically optimal temperatures of an organism can be decoupled from ecologically relevant temperatures [13]. For instance, locomotor performance tends to decrease drastically when body temperatures rise even slightly above physiologically optimal levels [14]. As a result, body temperatures below the physiological optimum may be more ecologically relevant, especially in fluctuating environments to ensure that overheating is avoided.

Active thermoregulation potentially permits activity in otherwise sub-optimal conditions. Thermoregulation relies on heat sources and sinks within a habitat to be able to maintain temperatures for physiological processes such as locomotion, assimilation, and growth [15]. Thermal sensitivity of performance permits us to categorize an organism as a thermal generalist or a thermal specialist. This thermal sensitivity ranges from a thermal generalist, which can perform over a broad range of temperatures, to a thermal specialist, whose performance is strongly dependent on temperature [10]. The interactions between thermal sensitivity and thermoregulation ultimately allow ectotherms to regulate their body temperature in concurrence with environmentally available temperatures to grow, survive, and reproduce [16]. Thus, changes in thermal conditions will potentially alter many aspects of activity and movement in ectotherms, including exploitation of available habitat [17], dispersal [18], and ultimately distribution [19].

The goal of the study was to analyze the ecological and physiological factors that influence daily activity and movement in box turtles throughout their active season using biologging devices carried by free-living animals. Overall dynamic body acceleration (ODBA) was used as a measure of box turtle activity, and the linear distance between consecutive GPS points or thread trailing were used as measures of movement. We analyzed the relationship between movement or accelerometer measurements with nearby weather-station data, determining the thermal sensitivity of activity, and analyzing domain and transition movement relative to activity. We hypothesized that (1) box turtle activity is thermally sensitive and thus temperature-dependent based on previous lab work, (2) box turtle movement and activity would be influenced by ambient conditions including precipitation and absolute humidity, and (3) fine-scale sampling methods will better reveal intensive movement while lower resolution sampling will be biased towards extensive movements.

## Methods

### Study site

We monitored Eastern box turtles (*Terrapene carolina carolina*) in Southwest Ohio at the Miami University Natural Areas (MUNA: 39.5° N, 84.7° W). For investigation of the environmental factors influencing activity and movement, box turtles are an ideal ectotherm to study because of their ability to tolerate heavy loads relative to their mass, allowing for multiple biologging devices to be attached. Our study sites are near the northern edge of their distribution east of the Mississippi river. Forest habitat in this landscape is highly fragmented due to the dominance of agriculture in the area, with forest fragments ranging in size from 5.5 ha to 400 ha. Climate in this region is characterized as humid continental with large seasonal temperature differences including warm to hot summers with high humidity and occasional severely cold winters, with precipitation distributed throughout the year [20]. Box turtles are listed as a species of special concern in Ohio with limited information on population demographics [21].

### Movement and activity monitoring

We monitored two groups of box turtles in 2014 and 2015 using a combination of thread trailing devices (group one) and GPS-Accelerometer tags (e-Obs, Grünwald, Germany; hereafter ‘GPS-ACC’, group two) which recorded both GPS locations and overall dynamic body acceleration (ODBA) data. Devices attached to the turtles were 83 × 26 × 20 mm (L x W x H) and were placed caudally on the shell such that the leading margin sloped upwards. Turtles monitored with GPS-ACC devices were all male (*n* = 12), and thread trailed turtles were both male (*n* = 7) and female (*n* = 4). We saw no difference between the sexes in our analyses and so data from thread trailing is presented as the combined data set. All box turtles were tracked with radio-telemetry using BD-52 transmitters (Holohil, Ontario, Canada) epoxied to the top of the shell for retrieval of devices.

Turtles in group one were tracked from May to July in 2014 and from June to July in 2015 using a methodology for thread trailing similar to Claussen et al. (1998) [22]. We epoxied a small plastic cylinder (height = 1.5 cm, diameter = 3 cm) to the posterior portion of the carapace that held a spool of nylon thread (228 m). Each turtle was released where it was first located and allowed a one-day acclimation period before beginning the trailing process. Each morning, individual turtles were located, and the thread tied to an anchor at the start point. We recorded the starting GPS at the beginning of each day using a Garmin 62 s handheld GPS (3 - 10 m resolution). Turtles were then located every 24-h to generate maps of daily movement. Turns were determined when the thread was caught on an object and changed direction. Each turn had a flag placed at that location and the series of flags permitted us to determine the turn angle and distance between each turn to the nearest centimeter and compass bearing to the nearest degree. Each turtle was monitored for up to 5 days, and days were omitted if the thread was broken or if the turtle moved beyond the capacity of the spool (45 out of 55 days yielded usable data). We replaced the thread as needed. Thread trailing data was then converted to Universal Transverse Mercator (UTM) coordinates in Zone 16S (WGS84, Ohio, USA). We used the initial starting coordinate from each day and converted the polar coordinates (bearing and distance) measured in the field to Cartesian coordinates (x, y-coordinates) and plotted the results to verify paths. UTM coordinates were then converted back to decimal degrees for analyses as appropriate.

Box turtles in group two were monitored during the 2015 field season from May until October. Turtles with the GPS-ACC devices also had temperature data loggers implanted internally (iButton DS1922L, see methods in Parlin et al. 2017 for details) which recorded body temperature at 5-min intervals throughout the study period. Box turtles in group two were monitored between 12 to 15 days. Loggers recorded GPS coordinates at 1-h intervals from 0700 to 1900, and accelerometer measurements recorded every 10 min (a 30-s burst) also between 0700 and 1900 h. All devices were calibrated prior to attachment and data was adjusted using device-specific calibrations. We had 1733 GPS observations for 12 turtles during the 2015 field season and 2337 thread trailing points converted to GPS coordinates for 11 turtles in 2014 and 2015 combined. All procedures followed approved MU Institutional Animal Care and Use Committee protocol (906) and complied with the Principles of Animal Care, publication no. 86–23, revised 1985, of the National Institutes of Health.

### Data analysis

All accelerometer measurements were converted into overall dynamic body acceleration (ODBA) prior to analysis. We used the average ODBA (± SE) at each degree Celsius of measured internal turtle body temperature to generate the data for testing non-linear splines to analyze intensity of activity as a function of body temperature. For later statistical analyses, we used the mean ODBA per hour to test across the units of measure of each relevant environmental variable. We characterized movement as extensive (larger mean turning angle and decreased step length) and intensive (smaller mean turning angle and increased step length) using three consecutive coordinates and two distances. Cluster analysis was used to characterize the turning angle and distance into each movement type for both GPS coordinates and thread trailing using the ‘ade4’ and ‘adehabitatLT’ package in R [23]. Movement was defined as the distance between two consecutive GPS coordinates and the turning angle was defined as the angle produced to reach the subsequent coordinate based on prior location.

We also derived a binary classification for each 30 s burst of acceleration measurement as either active and inactive for each individual. This was achieved by power transforming ODBA (Eq. 1 $x={ODBA}^{-\frac{1}{3}}$) and then fitting to a mixture distribution of two Gaussian distributions (Eq. 1). The assumption hereby is that the single ODBA measurements represent in their sum inactivity and activity resulting in a distribution consisting of two mixed Gaussian distributions each with an estimable mean (μ_a_ and μ_b_) and variance (σ_a_ and σ_b_ in eq. 1) (for each individual). Based on a non-linear least-squares approach (using the package nlsr in R version 3.4.3) we fitted the probability distribution function estimating both means and variances for each individual [24].

1 $$ pdf(y)=\frac{1}{\sqrt{2\pi {\sigma}_a^2}}\dot{e^{\frac{-{\left(x-{\mu}_a\right)}^2}{2{\sigma}_a^2}}}+\frac{1}{\sqrt{2\pi {\sigma}_b^2}}\dot{e^{\frac{-{\left(x-{\mu}_b\right)}^2}{2{\sigma}_b^2}}} $$

Using the estimated means and variances of the two Gaussian distributions, we then estimated the probability for each burst belonging to either of the two distributions (active and inactive) based on a probability density function for single Gaussian distributions with the estimated means and variances. This classification is independent of sampling and individual differences, which allows cross comparability among all individuals in the study (for the complete R code used see Additional file 1).

After defining ODBA values as active or inactive, we determined the proportion of activity (%) by counting the total number of active ODBA values per hour and dividing by the number of recordings taken during that hour. We analyzed the proportion of activity using a logistic regression test for differences between percentage groupings with turtle ID as a random effect, and analyzed comparisons using least-squares means.

### Statistical analysis

We then generated multiple non-linear splines and compared the equations using AIC to determine the best model fit. We then used the equation with the lowest AIC score for the thermal performance curves (TPC). We analyzed distance between GPS coordinates in relation to ecological factors from a nearby weather station (Butler County Regional Airport-Hogan Field weather station, Hamilton, OH, USA). We used stepwise regression (backward deletion) analysis to compare distance moved by box turtles and hourly ODBA with our predictor variables: *ambient temperature (°C)*, *absolute humidity (g/m*^*3*^*)*, *precipitation*, *cloud cover*, *time of day*, and their interactions. As interactions did not improve the model, they were not used in subsequent analyses. Predictor variables were based on previous natural history studies on box turtles and were consolidated to the most pertinent variables that could have an impact on box turtle activity and movement. Mixed-effect models of predictor variables as fixed effects and turtle ID as a random effect were tested against a null model using a likelihood ratio test [25]. We also compared the effect of slope on distance moved and intensity of activity with mixed effect models using turtle ID as a random effect and slope as a fixed effect. We did not use the distance between GPS coordinates from 1900 and 0700 h the next day as box turtles were sometimes active before our devices began recording. Distance data were natural log-transformed to meet assumptions of normality. Analysis of distance moved and percentage of activity, based on a gaussian distribution to define active and inactive ODBA values, was done using a least-squares regression analysis. For cluster analysis using GPS coordinates, we compared ODBA values associated with either extensive or intensive with a student’s *t*-test. We also used student’s *t*-test to compare distance moved and activity in the presence of precipitation. Data were analyzed in R version 3.0.2 [26], and in all cases α was set at 0.05.

## Results

### Thermal performance analysis

**Fig. 1:**
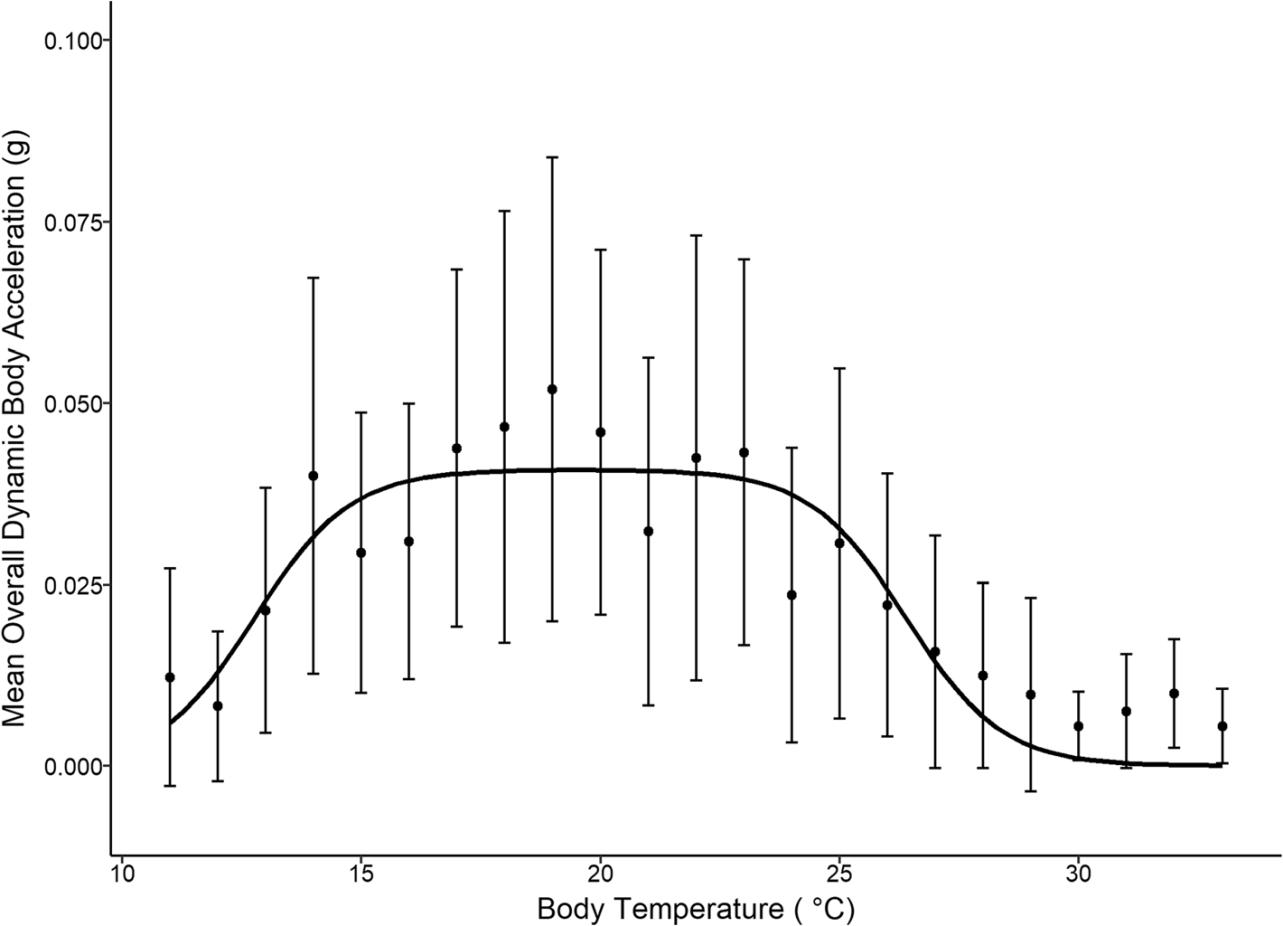
Thermal performance curve (TPC) of box turtle turtles (*n* = 12) monitored in 2015. Non-linear equation was fit to the mean overall dynamic body acceleration (ODBA) values. The black line represents the best fit regression. Box turtles had a relatively constant performance from 14 to 23 °C. Performance as a function of body temperature was not thermally dependent across this range giving box turtles a wide performance breadth

We determined the relationship between the intensity of activity, measured as ODBA, as a function of the body temperatures experienced by the turtles in the field. Body temperature measurements for box turtles ranged between 11.0 and 36.0 °C, and very few temperatures were measured below 12 or above 30 °C. The thermal performance curve (TPC) based on field data showed a broad, nearly uniform, performance between 14 and 23 °C (Fig. 1). The wide relationship between intensity of activity and body temperature indicates that box turtles are thermal generalists, and thus the performance of free-living box turtles is not dependent on temperature.

### Environment and activity analysis

**Fig. 2:**
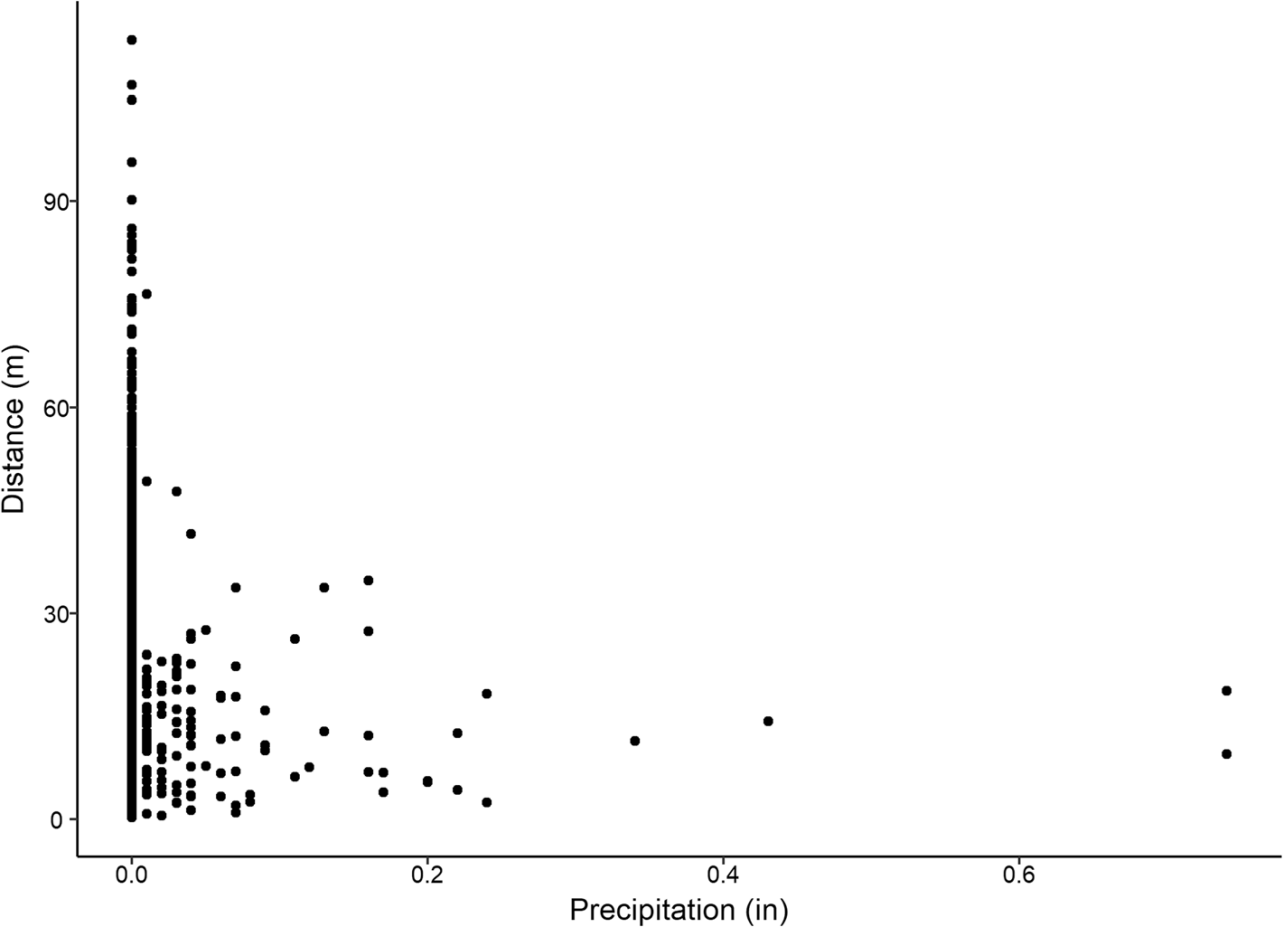
Distance moved as a function of precipitation measured by a nearby weather station. Mixed-effect model indicated precipitation and time of day as the best predictors of distance moved with a marginal r^2^ of only 0.008 based on the fixed effects of precipitation and time of day and a conditional r^2^ of 0.076. Although including the turtle ID as a random effect increased the r^2^, more than 90% of the data remained unexplained by the mixed-effect model. Precipitation was only recorded during 135 of the hour-intervals where movement occurred in box turtles

Linear mixed effect model analysis of microclimate variables including *precipitation*, *ambient temperature*, *cloud cover*, *time of day*, and *humidity*, indicated that *precipitation* (estimate = − 0.197; CI = − 0.279 – − 0.115) and *time of day* (estimate = 0.00293; CI = − 0.0266 – -0.0135) had the most influence on turtle movement (χ^2^_2_ = 8.7552, *p* = 0.01256) with a marginal r^2^ of only 0.008 based on the fixed effects of *precipitation* and *time of day* and a conditional r^2^ of 0.076 when also incorporating the random effect of turtle ID. We found that box turtles move 13 m each hour after it rains compared to the average of 17 m each hour when not raining (Fig.2, *t*_171_ = 3.02, *p* = 0.002). We had previously reported that box turtles move more during the morning than in the evening [12], although the differences were small. As box turtles are influenced by geotaxis, we tested for an effect of slope on movement, but found no significant influence on distance moved (χ ^2^_1_ = 2.63, *p* = 0.104). The regression analysis shows that more than 90% of the variation in the distance moved is unexplained by the model.

**Fig. 3:**
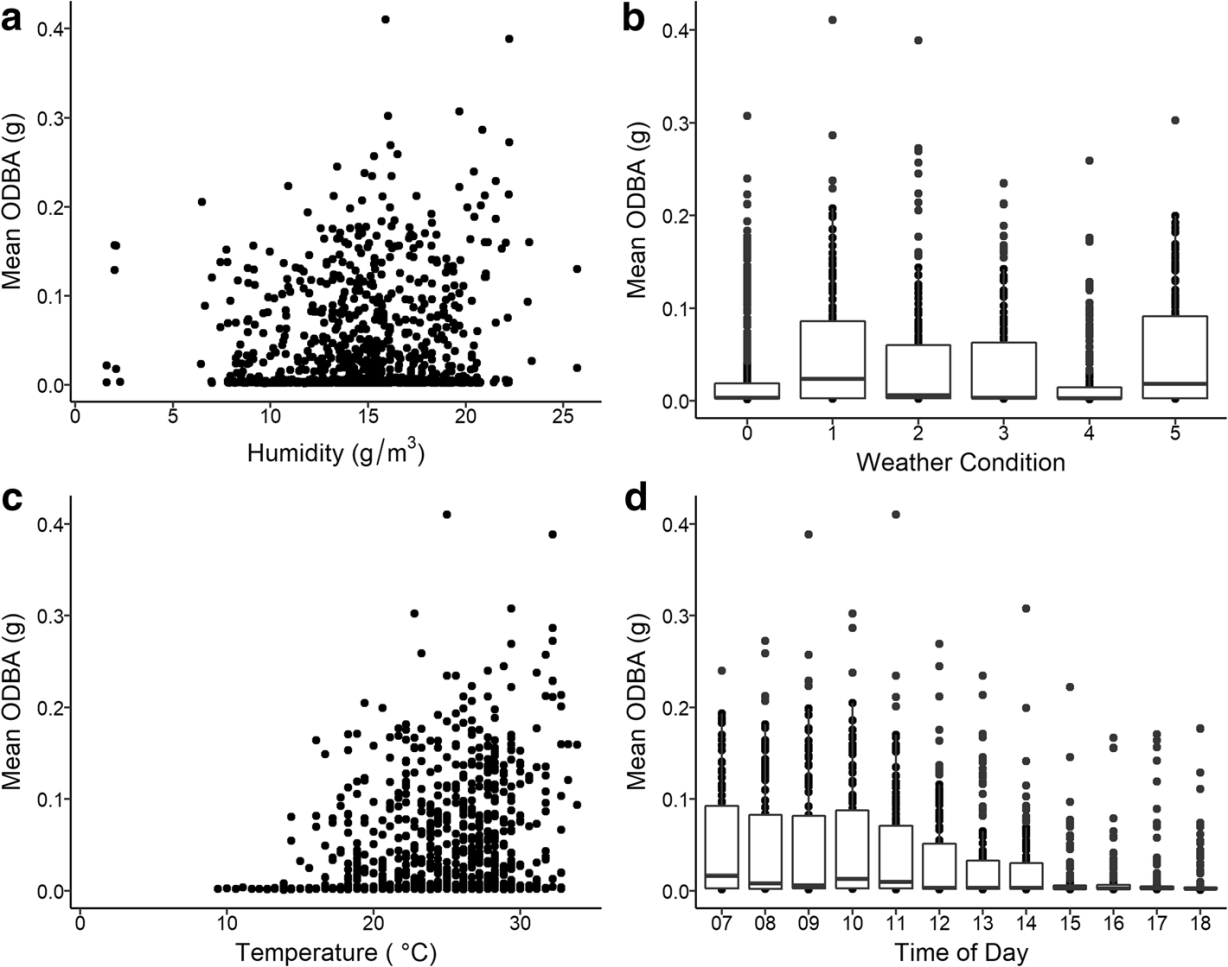
Climatic variables from the best model that had a significant influence on mean overall dynamic body acceleration (ODBA) including **a** absolute humidity, **b** weather condition, **c** temperature, and **d** time of day. Note that weather condition, classified by cloud cover, was split into five categories as follows: 0 = clear, 1 = scattered clouds, 2 = partly cloudy, 3 = mostly cloudy, 4 = overcast/haze, and 5 = light rain/thunderstorms. Intensity of activity as a function of climatic variable had a slight positive correlation with humidity and ambient temperature recorded. Mean ODBA decreased as time of day progressed from morning until the evening. The effect of clouds and rain did not follow a clear pattern

Box turtle activity was most influenced by *ambient temperature* (estimate = 0.000135; CI = 0.0009376–0.0017784), *absolute humidity* (estimate = 0.00291; CI = 0.00238–0.00345), *cloud cover* (estimate = 0.00229; CI = 0.00138–0.0032), and *time of day* (estimate = − 0.00373; CI = − 0.00419 – -0.00326) as significant (χ^2^_4_ = 181.02, *p* = 2.2e-16) with a marginal r^2^ of 0.147 based on fixed effects and a conditional r^2^ of 0.198 when also incorporating the random effect of the turtle (Fig. 3). We also found no significant difference in turtle activity in the presence or absence of *precipitation* (*t*_124_ = − 0.76004, *p* = 0.44) or for an effect of slope (χ ^2^_1_ = 2.63, *p* = 0.90). However, *precipitation* was only observed during 135 of the 1596 one-hour intervals when turtles were monitored. We additionally analyzed box turtle activity state as a percentage likelihood of activity during each hour interval and found that *ambient temperature*, *absolute humidity*, *cloud cover*, and *time of day* (χ^2^_4_ = 48.962, *p* = 5.9e-10) had similar significant effects although the marginal r^2^ was 0.076 and the conditional r^2^ was 0.108. Thus, around 80% of the variation in the intensity of activity and about 90% of the variation in the likelihood for being active remains unexplained by either model.

### Movement and activity analysis

We categorized coordinates for GPS and thread trailing into extensive and intensive movement to compare continuous micro-scale and hour interval macro-scale resolution. Comparison of sampling resolutions using thread trailing (micro-scale) and GPS-coordinates (macro-scale) yielded opposite characterizations of extensive and intensive movement (Table 1). Cluster analysis using GPS locations assigned 36% of the movement as extensive, and 63% of the movement as intensive. For the thread trailing data, 79% of the values were characterized as extensive movement and 21% of the values as intensive. Mean turning angle for both movement modes were higher for the thread trailing data than the GPS coordinates (Table 1).

**Table 1:** Total coordinates recorded for GPS and thread trailing monitoring techniques and subsequent classification of extensive and intensive movement counts using cluster analysis

Technique	Total Coordinates	Extensive	Intensive	Mean Turn Angle Extensive	Mean Turn Angle Intensive
GPS	1733	581	1002	128.2 ± 8.1	31.8 ± 6.7
Thread Trailing	2337	1837	474	150.8 ± 4.8	81.2 ± 9.7

Cluster analysis for GPS locations had 36% of the recordings characterized as extensive, and 63% of the recordings as intensive. For the thread trailing data, extensive movement was characterized as 79% of the values and intensive as 21% of the values. Mean turning angle was larger in the thread trailing for both the extensive and intensive movements

**Fig. 4:**
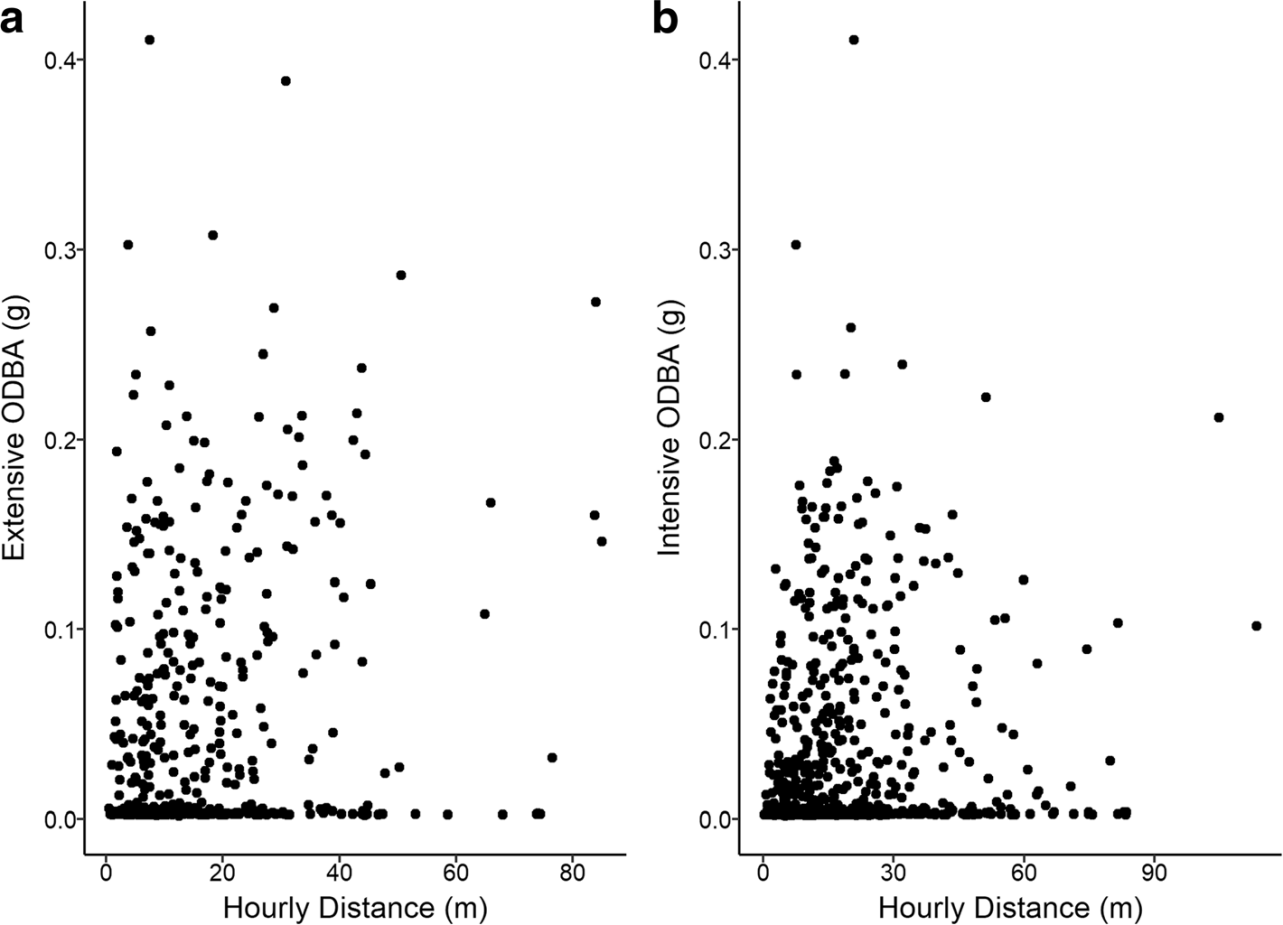
Mean hourly distance moved as a function of mean overall dynamic body acceleration (ODBA) for **a** extensive movement and **b** intensive movement categorizations. There was no correlation between distance moved and activity for either extensive or intensive movements. However, mean ODBA was significantly higher during extensive movement (0.049 g) than intensive (0.026 g), mainly due to a decreased likelihood of activity in the latter category (see Additional file 2)

We then separated all the ODBA values into two distributions as active (0.0110–1.387 g) and inactive (0.0011–0.01085 g). Using this distribution, we analyzed the intensity of activity for each category of movement and found that intensity of activity for either movement mode had no correlation with the distance moved (Fig. 4). Although maximal values recorded for extensive and intensive movement were similar, we found the mean intensity of activity to be significantly higher for movements characterized as extensive (Fig. 4a, 0.046 g) than for those characterized as intensive (Fig. 4b, 0.026 g, t_767_ = 6.1192, *p* < 0.05), mainly due to a decreased likelihood of activity for intensive movement characterization. Although there was no relationship between the intensity of activity and movements, we found a significant relationship between the likelihood of activity in each hour and distance moved (F_6_ = 5.5994, *p* < 0.05, Additional file 2), such that the mean distance moved varied from 16.1 to 24.6 m as the likelihood of activity increased.

## Discussion

Eastern box turtles are a species of special concern across most of their range with worrying population declines reported. One of the reasons for this decline is linked in part to habitat loss [21]. As habitats for box turtles are lost and other human impacts increase, we may expect turtle movement to decrease similarly to that of mammals as recently reported [27]. We had previously reported that the body temperature of box turtles was unrelated to the distances moved. We thus sought to expand this observation to determine what, if any, environmental factors may dictate turtle movement, based on prior work done on box turtles. Further, the determinants of the intensity of activity, as measured by 3-dimensional accelerometry, may also give insight into the movement ecology of free-living turtles as they interact with their environment. Previous work indicated that box turtles exist within a narrow range of microclimate variables [28], become more active if the temperature drops and rain begins to fall due to thunderstorms [29], and have their peak activity during the morning and become relatively inactive during the evening [30]. Our results did not support the hypothesis that movement is thermally sensitive. While there was a significant relationship between movement and several climatic variables, the strength of these were weak. Thus, other factors, such as feeding, hydration state, mate seeking or vegetation structure may prove to be more important determinants of turtle movement and activity.

The recent advancement of biologging technology, allowing for continuous monitoring for extended periods of time, permits a more in depth understanding of box turtle life history. Our analysis based on these relevant environmental parameters showed no relationship between movement and *ambient temperature*, *absolute humidity* or *cloud cover*, while revealing significant impacts of both time of day and precipitation on movement. However, these only explained about 10% of the variation in distance moved. Thus, in several important ways, our data did not agree with these previous studies as we found that the factors measured did have an impact on box turtle movement but often in an opposite direction. For example, similar to our previous work [12], we found no effect of temperature, and we found that precipitation decreased movement in our population of box turtles, unlike Webb (1963) [29].

Intensity of activity, measured as ODBA, was best explained by *ambient temperature*, *absolute humidity*, *cloud cover*, and *time of day*, while *precipitation* was not significant. Similar to the extent of movement, these significant environmental factors only explained around 20% of the variation in activity. The environmental factors influencing the likelihood of activity, measured as a percentage of recording categorized as active per hour, also only explained around 10% of the variation in hourly activity. This fits well with our observation that the thermal reaction norm showed that the intensity of activity was nearly unchanged across a very broad range of body temperatures. As we previously showed that box turtles are thermal generalists, with body temperatures similar to ambient [12], it comes as no surprise that the ambient temperature also had little effect on activity. This work further supports the conclusion that box turtle movement and daily activity are highly resilient in response to climate conditions.

Given that we used weather station data, it is possible that the differences between those data and micro-climates selected by turtles could alter these relationships. It is also plausible that activity is driven by prior exposure to the landscape. Box turtles have an incredible ability to navigate back towards their home range when displaced and are even capable of moving at night [31]. Further research monitoring the activity and movement of displaced turtles using biologging technology would provide added insight into the capability of these ectotherms to navigate their habitat, especially in fragmented landscapes.

Unlike previous laboratory work of temperature-dependent movement on reptiles [8–10] including box turtles [11], we found that ambient and body temperature were generally irrelevant as determinants of both activity and movement. This suggests that laboratory measures of maximal locomotor capacity may be erroneous for most movement in nature, at least for turtles. Laboratory conditions may not represent patterns and processes observed in the field, thus our data point to the importance of studies of free-living animals to best delineate the factors that determine the realized performance. As box turtles were able to be active at all temperatures observed in the field, other factors presumably act to determine the extent moved. However, temperature is a driving force for ectotherms [10], dictating many physiological functions, one of which may supersede locomotion.

To better understand the nature of box turtle movement within a habitat patch, we categorized each step as intensive (foraging) or extensive (exploratory). As resolution of the data collection method could influence this analysis [5, 32], we compared movement data collected with animal borne GPS loggers (with 1-h intervals) to data from continuous thread trailing. The outcomes from this analysis returned nearly opposite interpretations, suggesting that when data were sampled at low resolution that turtles are primarily foraging, while when sampled at high resolution that turtles are primarily engaged in exploratory movement. However, given that we did not simultaneously monitor GPS location and thread trailing, direct comparison between movement measurements in foraging sites remains unclear and caution must be taken with interpretation of data recorded at different scales.

This data support and extend our earlier observation that Eastern box turtles are a thermoconforming ectotherm, with movement that is nearly identical across an impressively wide range of body temperatures [12]. Although the extent of movement and intensity of activity are significantly affected by several environmental factors, none do so with great explanatory power. Thus, we conclude that in addition to being thermal generalists, the movement ecology of Eastern box turtles is largely independent of the habitat characteristics examined. Movement patterns in Galapagos tortoises are driven by changes in vegetation [33] and similar constraints may be relevant for box turtles. Although we frequently observed turtles feeding and they tended to congregate in a region of the study area with a high density of mulberry trees (*Morus* spp.), we lack detailed data on feeding habits. Generally, box turtle movement appeared to be haphazard within each forest patch and we documented no box turtles traversing from one habitat fragment to another. Finally, given the marked difference between the categorization of movements as intensive (foraging) or extensive (exploratory) depending on the method used to record movements, further comparisons of fine-scale and coarse-scale movement, undertaken with a more uniform methodology, may be important for future studies.

## Conclusions

This study contributes to understanding the interaction between physiology and movement, and the effects of climate conditions on activity in the field. Our results also show the importance of difference in fine-scale sampling resolution compared to coarse-scale resolution for characterizing and analyzing movement. Although laboratory studies have shown a strong thermal dependence of physiological performance, field monitoring can decouple the ecologically relevant temperatures from the physiologically optimal. This provides further insight into the patterns and processes observed in the field for free-living individuals and can further our understanding of how changes in climate conditions can impact a species.

## Additional files

Additional file 1: R Script for binary classification of ODBA values from accelerometer data. (R 6 kb)

Additional file 2: Additional figure of proportion of activity and distance moved. (DOCX 26 kb)

## References

[1] Caldwell IR, Nams VO (2006). A compass without a map: tortuosity and orientation of eastern painted turtles (*Chrysemys picta picta*) released in unfamiliar territory. Can J Zool.

[2] Malishev M, Bull CM, Kearney MR. An individual-based model of ectotherm movement integrating metabolic and microclimatic constraints. Methods Ecol Evol. 2017; 10.1111/2041-210X.12909.

[3] Sears MW, Angilletta MJ, Schuler MS, Borchert J, Dilliplane KF, Stegman M, Rusch TW, Mitchell WA (2016). Configuration of the thermal landscape determines thermoregulatory performance of ectotherms. PNAS.

[4] Gunderson AR, Leal M (2012). Geographic variation in vulnerability to climate warming in tropical Caribbean lizard. Funct Ecol.

[5] Nams VO (2005). Using animal movement paths to measure response to spatial scale. Oecologia.

[6] Stickel LF (1950). Populations and home range relationships of the box turtle, Terrapene c. Carolina (Linnaeus). Ecol Monogr.

[7] Price-Rees SJ, Lindström T, Brown GP, Shine R (2014). The effects of weather conditions on dispersal behaviour of free-ranging lizards (Tiliqua, Scincidae) in tropical Australia. Funct Ecol.

[8] Jayne BC, Bennett AF, Lauder GV (1988). Effects of temperature on muscle-activity during lizard locomotion. Am Zool.

[9] Rome LC, Bennett AF (1990). Influence of temperature on muscle and locomotor performance – introduction. Am J Phys.

[10] Angilletta MJ. Thermal adaptation: a theoretical and empirical synthesis. New York: Oxford University Press; 2009.

[11] Adams NA, Claussen DL, Skillings J (1989). Effects of temperature on voluntary locomotion of the eastern box turtle, *Terrapene carolina carolina*. Copeia.

[12] Parlin AF, do Amaral JPS, Kelly Dougherty J, Stevens MHH, Schaeffer PJ (2017). 2017. Thermoregulatory performance and habitat selection of the eastern box turtle (Terrapene carolina carolina). Conserv Physiol.

[13] Huey RB, Hertz PE (1984). Is a Jack-of-all temperatures a master of none?. Evolution.

[14] Martin LT, Huey RB (2008). Why “suboptimal” is optimal: Jensen’s inequality and ectotherm thermal preferences. Am Nat.

[15] Huey RB (1991). Physiological consequences of habitat selection. Am Nat.

[16] Huey RB, Stevenson RD (1979). Integrating thermal physiology and ecology of ectotherms: a discussion of approaches. Am Zool.

[17] Kearney M (2013). Activity restriction and the mechanistic basis for extinctions under climate warming. Ecol Lett.

[18] Kearney M, Porter W (2009). Mechanistic niche modelling: combining physiological and spatial data to predict species’ ranges. Ecol Lett.

[19] Buckley LB, Tewksbury JJ, Deutsch CA (2013). Can terrestrial ectotherms escape the heat of climate change by moving?. Proc Roy Soc B.

[20] Karl TR, Koss WJ. Regional and National Monthly, Seasonal, and Annual Temperature Weighted by Area, 1895–1983. Hist Climatol Ser. 1984;4:1–38.

[21] van Dijk PP. Terrapene carolina.(errata version published in 2016). The IUCN Red List of Threatened Species 2011. T21641A97428179. Accessed on 21 Mar 2018

[22] Claussen DL, Finkler MS, Smith MM (1997). Thread trailing of turtles: methods for evaluating spatial movements and pathway structure. Can J Zool.

[23] Calenge C (2006). The package “adehabitat” for the R software: a tool for the analysis of space and habitat use by animals. Ecol Model.

[24] Nash JC, Murdoch D. (2018). Nlsr: functions for nonlinear least squares solutions. R package version. 2018;1:28. https://cran.r-project.org/package=nlsr. Accessed 5 July 2018.

[25] Bates D, Maechler M, Bolker B, Walker S. Fitting linear mixed-effects models using lme4. J Stat Softw. 2015; 10.18637/jss.v067.i01. Accessed 5 July 2018.

[26] R Development Core Team. R: A language and environment for statistical computing. Vienna: R Foundation for Statistical Computing; 2015. ISBN 3–900051–07-0, URL https://www.r-project.org. Accessed 5 July 2018.

[27] Tucker MA, Böhning-Gaese K, Fagan WF, Fryxell JM, Van Moorter B, Alberts SC, Ali AH, Allen AM, Attias N, Avgar T, Bartlam-Brooks H (2018). Moving in the Anthropocene: global reductions in terrestrial mammalian movements. Science.

[28] Reagan DP (1974). Habitat selection in the three-toed box turtle, Terrapene carolina triunguis. Copeia.

[29] Webb RG, Minckley WL, Craddock JE (1963). Remarks on the Coahuilan box turtle, Terrapene coahuila (Testudines, Emydidae). Southwest Nat.

[30] Dodd CK. North American box turtles: a natural history: University of Oklahoma Press; 2002.

[31] Metcalf E, Metcalf AL (1970). Observations on ornate box turtles (Terrapene ornata ornata Agassiz). Trans Kans Acad Sci (1903-).

[32] Benhamou S (2004). How to reliably estimate the tortuosity of an animal’s path: straightness, sinuosity, or fractal dimension?. J Theor Biol.

[33] Yackulic CB, Blake S, Bastille-Rousseau G (2017). Benefits of the destinations, not costs of the journeys, shape partial migration patterns. J Anim Ecol.

