# Supplementary material for: Activity and movement of free-living box turtles are largely independent of ambient and thermal conditions
Source: Mov Ecol. 2018 Jul 19;6:12. doi: 10.1186/s40462-018-0130-8 (PMC6052674; doi:10.1186/s40462-018-0130-8)
Supplement: Supplementary file 2 — Additional figure of proportion of activity and distance moved. (DOCX 26 kb) [file 40462_2018_130_MOESM2_ESM.docx]

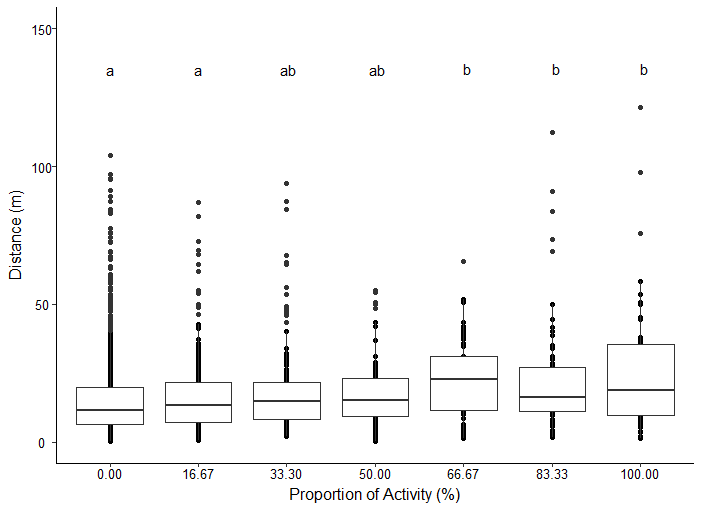


Figure S1. Percent of recordings (proportion of activity) characterized as an active state in box turtles during distance moved. Mean distance moved varied between 16.1 (0% active state) to 24.6m (100% active state) with a wide spread in the data during all categorizations of activity in an hour. Proportion of activity during any given hour had a significant effect on the distance moved during that hour and least-squares means for likelihood of activity had overlapping similarities (denoted by letters) from 0.00% to 50.0% and 33.00% to 100% activity for distance as a function.
